# Brivaracetam in treating epileptic encephalopathy and refractory focal epilepsies in patients under 14 years of age

**DOI:** 10.22037/ijcn.v15i4.29819

**Published:** 2021

**Authors:** Angelo RUSSO, Vittoria CUTERI, Lalit BANSAL, Paolo BONANNI, Alberto DANIELI, Antonella PINI, Giuseppe GOBBI

**Affiliations:** 1 Pediatric Neurologist and Psychiatrist - Epileptologist IRCCS, Institute of Neurological Sciences of Bologna Bellaria-Maggiore Hospital and Sant'Orsola University Hospital, Bologna Child Neurology Unit - Seizure Unit Bologna, Bologna, Italy; 2 Child Neurology and Psichiatric Unit, Departement of Medical and Surgical Science (DIMEC), S.Orsola Hospital, University of Bologna, Bologna, Italy.; 3 Division of Neurology, Children’s Mercy Hospital, University of Missouri Kansas City, Missouri, United States.; 4 l’IRCCS “Medea” - La Nostra Famiglia di Conegliano, U.O.C Epilessia e Psicopatologia, Italy; 5 IRCCS, Institute of Neurological Sciences of Bologna, Bologna, Italy

**Keywords:** Brivaracetam, Childhood epilepsy, Drug-resistant epilepsy, Epileptic encephalopathy

## Abstract

**Objectives:**

To analyze the efficacy and safety of Brivaracetam in pediatric patients with epileptic encephalopathy or unresponsive focal epilepsy.

**Materials & Methods:**

This retrospective study included eight pediatric patients with EE or unresponsive focal epilepsy. Inclusion criteria: (1) ≤14 years, (2) history of refractory epilepsy, (3) at least one month of continuous therapy with BRV, and (4) at least six months of follow-up. Exclusion criteria: (1) variation of concomitant antiepileptic drugs during the previous and/or subsequent four weeks of the BRV introduction, (2) levetiracetam in therapy, (3) epilepsy secondary to the progressive cerebral disease, tumor, or any other progressive neurodegenerative diseases, and (4) a status epilepticus a month before screening or during the baseline period. The efficacy of BRV was defined as ≥50% of seizure frequency reduction at the end of the follow-up, compared to baseline.

**Results:**

All patients showed ≥50% seizure frequency reduction, of whom 37.5% were seizure-free, 25% had a frequency reduction of ≥75%, and 37.5% had frequency reduction of ≥ 50%. All patients with an epilepsy onset >12 months and epilepsy duration of ≤6 years were seizure-free. The maximum effect was achieved at 2 mg/kg/day, and focal seizures revealed a better response than epileptic encephalopathy. A remarkably positive effect of the Brivaracetam was noticed in patients with encephalopathy regarding the status epilepticus during sleep; however, no relevant side-effects were noted.

**Conclusion:**

Brivaracetam was an effective and well-tolerated treatment in pediatric patients with epileptic encephalopathy or unresponsive focal epilepsy, especially for the epilepsy onset >12 months and the epilepsy duration ≤6 years. The total effect was not dose-dependent. Brivaracetam could represent an indication of encephalopathy regarding the status epilepticus during sleep.

## Introduction

In the last 20 years, due to the high prevalence of therapy-resistant epilepsy, there has been a need to develop newer antiepileptic drugs with a more recent mechanism of action such as synaptic vesicle glycoprotein 2A, a transmembrane glycoprotein, and galactose transporter (1- 4).

Brivaracetam is a selective, high-affinity ligand for synaptic vesicle glycoprotein 2A, approved as adjunctive therapy and monotherapy for the treatment of focal seizures in patients aged ≥4 years in the United States and for focal seizures with or without secondary generalization in patients aged ≥4 years in the European Union (5, 6). Several randomized controlled trials have been conducted in the adult population, revealing promising data on the efficacy and tolerability of Brivaracetam with ≥50% responder rate (7-13).

The results obtained from pre-marketing trials were confirmed by subsequent postmarketing studies addressing adult patients or a mixed population with a small group of pediatric subjects with different epileptic conditions, including unresponsive focal epilepsy, refractory status epilepticus (14-21), generalized epilepsies, absence status epilepticus (22), and epileptic encephalopathies (23). The literature is limited on exclusively pediatric population with unresponsive epilepsy, and the existing data come from a phase IIA study and a retrospective study (24, 25). In both studies, adjunctive treatments with Brivaracetam showed a similar responder rate as observed in previous studies on adult subjects. However, in the phase IIA trial (24), the Brivaracetam therapy was administered for only three weeks, and neither was the etiology of focal epilepsy specified nor were the drug-resistant and non-drug-resistant patients separated. In the postmarketing study (25), most patients (77%) were currently or previously treated with Levetiracetam.

Given the gap in the literature and the need for better documentation of the effectiveness and safety of Brivaracetam in children, we conducted this retrospective review to analyze the effectiveness and tolerability of the adjunctive therapy with Brivaracetam in pediatric patients with epileptic encephalopathy and unresponsive focal epilepsy.

## Materials & Methods

In this study, eight patients with epileptic encephalopathy or unresponsive focal epilepsy were retrospectively selected from a cohort of 25 pediatric patients, for whom Brivaracetam was used as an add-on therapy. All patients were followed up in the IRCCS Neurological Science Institute of Bologna and at IRCCS “Medea” La Nostra Famiglia of Conegliano from November 2018 to July 2019.

The clinical diagnosis of epilepsy was supported using an electroencephalogram and brain MRI imaging. Inclusion criteria were as follows: 1) ≤ 14 years; 2) history of refractory epilepsy; 3) at least one month of continuous therapy with Brivaracetam, and 4) at least six months of follow-up. Exclusion criteria were (1) variation of concomitant AEDs during the previous and/or subsequent four weeks of the Brivaracetam introduction, (2) concomitant therapy with Levetiracetam, (3) epilepsy secondary to progressive the cerebral disease, tumor, or any other progressive neurodegenerative disorders, and (4) a history of status epilepticus in the month before the baseline period or during the follow-up phase. 

All patients began Brivaracetam at 1 mg/kg/day. According to the clinical response, Brivaracetam was increased by 1 mg/kg/day, with a maximum dosage of 4 mg/kg/day. Treatment with Brivaracetam was discontinued in none of the patients. 

We collected the following clinical data: age at epilepsy onset, duration of epilepsy before the Brivaracetam, etiology, seizure and epilepsy type, and seizure frequency. Seizure types and epilepsy syndromes were classified in accordance with the International League Against Epilepsy classification of epileptic seizures and epileptic syndromes (26,27). Seizure frequency was obtained from medical notes and seizure diaries. To this end, seizure frequencies were averaged and recorded monthly for the last three months at baseline and the end of the follow-up. Seizure frequency was classified as follows: daily (≥ 1 seizure per day) and weekly (≥ 4 seizures per month, ≤6 seizures per week) seizure; age at epilepsy onset as ≤ 12 months and > 12 months, seizure frequency as ≤weekly and daily, and epilepsy duration before Brivaracetam as ≤ 6 years and > 6 years. The efficacy of Brivaracetam was defined as ≥50% of seizure frequency reduction at the end of the follow-up phase compared to baseline (1 month before Brivaracetam), and the patients were considered as good responders if the seizure reduction was **≥** 50% and very good responders if it was ≥ 75%. Seizure freedom in the present study was defined as complete freedom from all seizures (seizure frequency of 0) during the last follow-up phase. 

We considered a dependent variable (namely age at epilepsy onset, duration of epilepsy before the Brivaracetam, etiology, seizure and epilepsy type, and seizure frequency) as “likely influencing” the independent variable (seizure frequency) if the difference between the two arms of the dependent variable was more than 25%.

According to the U.S. Food and Drug Administration definitions (28) the appearance of adverse events was also checked. Patients or their legal guardians submitted written informed consent.

## Results

Of the twenty-five patients initiating Brivaracetam therapy, eight met our inclusion/exclusion criteria; however, none of the patients withdrew from the treatment. 

Table 1 summarizes the participants’ characteristics at the baseline and at the end of the follow-up phase. The study population included four males and four females. The median age of this cohort was 13 years (ranging from eight years and four months - 14 years), and their median age for epilepsy onset was seven months (ranging from 1- 96 months). Four patients (4/8, 50%) had unresponsive focal epilepsy, and four patients (4/8, 50%) had epileptic encephalopathy. Among patients with unresponsive focal epilepsy, there were two cases of structural etiology (focal cortical dysplasia), one case with genetic etiology (encephalopathy related to status epilepticus during sleep), and one with unknown etiology. Of subjects with epileptic encephalopathy, three patients had structural etiology (2 Lennox-Gastaut syndromes and one unknown EE), and one patient had genetic etiology (Dravet syndrome).

Intellectual disability was reported in 62.5% of the patients (N = 5/8). At the baseline, four patients reported the daily frequency of seizures (50% of subjects), four subjects the weekly frequency of seizures (50% of subjects). The median dose of add-on Brivaracetam was 3.5 mg/kg/day (range 2–4 mg/kg/day). At the end of the follow-up phase, all patients (100%) continued the Brivaracetam therapy. The mean number of previously-used AEDs was 10 (range 3–14), while the mean number of concomitant antiepileptic drugs was 2.3 (range 2–4). 

**Figure 1:**
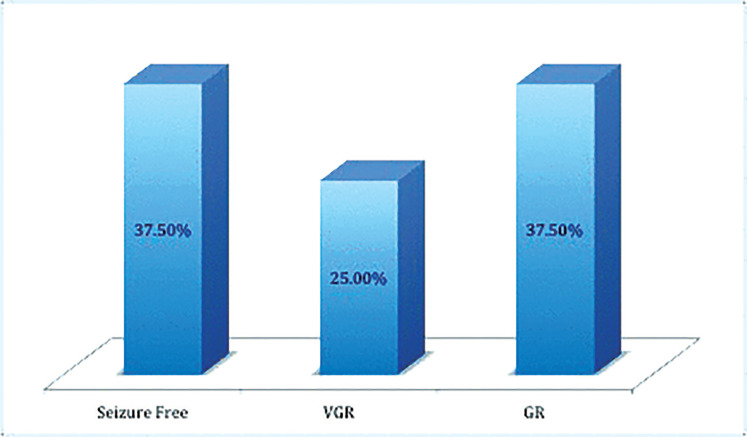
Brivaracetam efficacy. VGR: very good responders (≥ 75% seizure reduction); GR: good responders (≥ 50% seizure reduction)

At a median 8-month follow-up (range 6–11 months), the overall response rate (≥50% seizure frequency reduction) was 100% (8/8 patients). Of the participants, three patients were seizure-free (37.5%), two cases had a frequency reduction of ≥ 75% (25%), and three patients had a frequency reduction of ≥ 50% (37.5%) (Fig. 1). In all patients, the maximum benefit of Brivaracetam was achieved at 2 mg/kg/day. No further benefit was reported when the daily dose was increased by 3 mg/kg/day and 4 mg/kg/day. 

**Figure 2:**
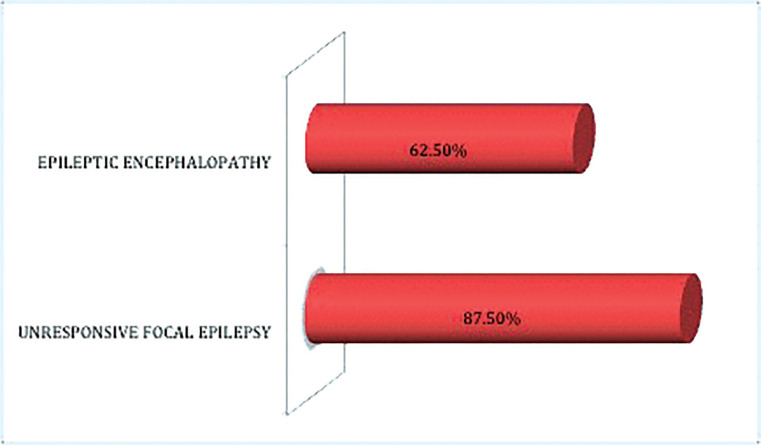
Brivaracetam responder rate (≥50% seizure reduction) based on type of epilepsy

As shown in Fig. 2, patients with focal seizures tended to respond better (response rate 87.5%) than the patients with epileptic encephalopathy (response rate 62.5%). 

As shown in Table 2, patients with an epilepsy onset > 12 months and duration of epilepsy ≤ 6 years were seizure-free at the last follow-up phase, compared to those with an epilepsy onset *≤* 12 months and duration of epilepsy > 6 years (responder rate of 60%).

No differences was observed between the seizure frequency before the Brivaracetam (response rate: ≤ weekly 81.25%, daily 68.25%) and etiology (response rate: genetic etiology 75%, structural etiology 81.25%). 

**Figure 3:**
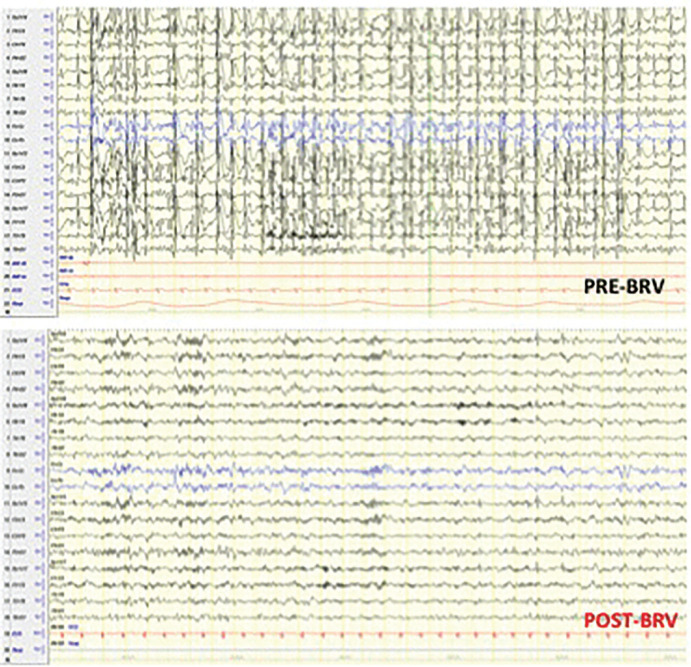
Sleep EEG in patient with encephalopathy regarding status epilepticus during sleep. Remarkable improvement could be observed after the therapy with Brivaracetam

In addition to the seizure-freedom, a quick and remarkable improvement of the electroencephalogram and behavior was noticed in a patient with encephalopathy regarding status epilepticus during sleep (Fig. 3). Relevant adverse events were reported in none of the patients. 

## Discussion

Although regulatory trials (7-13) and postmarketing studies (14-23) have confirmed Brivaracetam as an effective and well-tolerated antiepileptic drug, the effects of Brivaracetam in pediatric patients are less-investigated, especially in the real-world clinical settings. 

Only two studies have exclusively addressed the pediatric population (24,25). The first was a phase IIA, open-label, single-arm, multicenter trial (24), and the second one was a retrospective multicenter study (25). In these two studies, the efficacy and tolerability of the adjunctive treatment with Brivaracetam were compared to the previous studies on the adult population, with a response rate of ≥50%. They reported the most common treatment-related adverse events as somnolence and decreased appetite; however, the studies had some bias.

In the phase II trial, the Brivaracetam was administered only for three weeks. Moreover, neither was the etiology of focal epilepsy specified, nor were the drug-resistant and non-drug-resistant patients separated. These limitations imply that their main goal was to characterize the steady-state plasma concentrations of Brivaracetam oral solution and its metabolites and not to address its efficacy and tolerability. Most of the patients (77%) were currently or previously treated with Levetiracetam in the postmarketing study. The authors concluded that prior Levetiracetam exposure was reported to be associated with lower responder rates, and that the tolerability of Levetiracetam might be a reason for the high retention (97% at three months) and reasonable responder rates in patients on Brivaracetam. 

Our study confirmed the efficacy of previous data showing a response rate > 50% (Fig. 1). The best response was observed in patients with an epilepsy onset > 12 months and duration of epilepsy ≤ 6 years (100% seizure-free) (Table 2). Moreover, better response was achieved in drug-resistant focal epilepsies (87.5%) as compared to patients with EE (62.5%) (Fig. 2). The most remarkable finding was the Brivaracetam response of the patient with encephalopathy regarding the status epilepticus during sleep, who became seizure-free with a quick and remarkable improvement in the electroencephalogram (Fig. 3) and behavior issues. The same finding has not been reported in the literature. A better response in unresponsive focal epilepsies than for EE was expected due to the greater severity of the latter. However, also in epileptic encephalopathy, our results were strongly positive and in line with the findings of a recent adult study on 44 patients with the mean age of 28.3 ± 14.5 years, suggesting Brivaracetam as an effective and well-tolerated antiepileptic drug in adult patients with epileptic encephalopathy (retention rate of 65% in three months, 52% in six months, and 41% in 12 months). 

Another notable result in our study was that the overall effect of the Brivaracetam was not dose-dependent, with a maximum efficacy of 2 mg/kg/day. In this regard, no similar or comparable data have been published yet. 

The main limitation of the present study was the retrospective nature of the research, with no control group and the inclusion of a small cohort of patients with unresponsive epilepsy of various etiologies. Another limitation was the relatively short follow-up phase, which did not allow the long-term outcome expectations.

On the other hand, the advantages of a retrospective study was that the findings cannot be predetermined, that the evaluations are based on existing data sources in which both exposure and outcomes are available, and that the findings cannot be tailored to the data collected for a specific drug. 

Prospective controlled studies on large homogeneous population are required to evaluate the long-term effectiveness and tolerability of Brivaracetam in pediatric patients with epilepsy.

**Table 1:** *The participants’ c* *linic and demographic characteristics*

	**N=8**
**Age (years)**	
*Mean ± SD*	11.9±2.5
*Median (range)*	13 (8.3-14)
**Gender, N (%)**	
*Males *	4 (50%)
*Females*	4 (50%)
**Age at onset of epilepsy (months)**	
*≤ 12 months*	5 (62.5%)
*> 12 months*	3 (37.5%)
**Duration of epilepsy (years)**	
*> 6 years*	5 (62.5%)
*≤ 6 years*	3 (37.5%)
**Type of epilepsy, N (%)**	
*Focal epilepsy*	4 (50%)
*Epileptic Encephalopathy*	4 (50%)
**Type of seizures, N (%)**	
*Focal*	4 (50%)
*Generalized*	4 (50%)
**Aetiology of epilepsy, N (%)**	
*Structural*	5 (62.5%)
*Genetic*	3 (37.5%)
**Frequency of seizures at baseline, N (%)**	
*Daily*	4 (50%)
*Weekly*	4 (50%)
**Intellectual disability, N(%)**	
*Yes*	5 (62.5%)
*No*	3 (37.7%)
**Number of AED**	
*Mean ± SD*	10 ± 3.3
*Median (range)*	10.5 (3-14)

**Table 2:** *Brivaracetam responder rate (* *≥50% seizure reduction)* *based on age at epilepsy onset and duration of epilepsy* *before* *Brivaracetam treatment*

**Number of patients**	**Age at epilepsy onset **	**Duration of epilepsy before Brivaracetam**	**Responder rate**
5	≤ 12 months	> 6 years	60%
**3**	**> 12 months**	**≤ 6 years**	**100%**

## Authors’ Contributions

A. R. was the key member of the clinical team, he had full access to all research data, was in charge of confirming the integrity of data and the accuracy of the data analysis, and wrote the manuscript. V. C. had full access to all research data, was in charge of confirming the integrity of data and the accuracy of data analysis, and wrote the manuscript. L. B., P. B., A. D., and A. P. revised the first draft of the manuscript. G. G. codesigned the study and discussed the interpretation of the data. All authors contributed, read, and approved the manuscript. 

## Conflicti of Interest

The authors declared no potential conflict of interests regarding the research, authorship, and/or publication of this article. 
